# F78. OVERCOMING A BOTTOM-UP ATTENTIONAL BIAS BY PROVIDING TOP-DOWN INFORMATION DURING WORKING MEMORY ENCODING IN SCHIZOPHRENIA

**DOI:** 10.1093/schbul/sby017.609

**Published:** 2018-04-01

**Authors:** Catherine Barnes, Lara Rösler, Michael Schaum, Deliah Macht, Benjamin Peters, Jutta Mayer, Michael Wibral, Andreas Reif, Robert Bittner

**Affiliations:** 1 Goethe University Hospital Frankfurt; 2 University of Würzburg; 3 Institute for Medical Psychology, Goethe University Hospital Frankfurt

## Abstract

**Background:**

Cognitive impairments including deficits in working memory are commonly observed in schizophrenia. A bottom-up attentional bias has been suggested for encoding visually salient yet irrelevant information. To date it is not known if this bias persists when additional top-down information in the form of a predictive cue is provided. We were motivated to clarify this issue.

**Methods:**

40 patients with schizophrenia were measured and matched with 40 healthy control participants. During a change detection task four Gabor patches (two flickering and two non-flickering) with varying orientations were shown and participants had to memorize the orientations of the Gabor patches. A colored fixation cross was displayed before the stimuli either cueing two (predictive cue) or all four (non-predictive cue) Gabor patch locations resulting in a 2 x 2 design of four conditions with the factors salience (flickering vs. non-flickering) and cue (predictive cue vs. non-predictive cue). During retrieval a single Gabor patch was displayed, and participants reported if the orientation was the same or had changed in that location. At the beginning of each block participants were instructed to either encode the flickering or non-flickering patches (targets) whose location could either be cued or uncued. In 80 % of trials, a target was probed during retrieval.

**Results:**

Patients encoded less information than healthy controls in all four conditions. Both healthy controls and patients encoded more visually salient information than non-salient information, and performance was near chance level during non-target trials. Patients encoded significantly more information when a predictive cue was provided before encoding visually non-salient information.

**Discussion:**

Patients were able to overcome their bottom-up attentional bias of encoding visually salient irrelevant information when provided with top-down information. These findings are in line with previous reports of a bottom-up attentional bias during working memory encoding in schizophrenia. We propose that this bias can be overcome by providing additional top-down information.

